# Paycheck Protection Program (PPP) COVID-19 relief funding for veteran-, minority-, and women- owned dental businesses: A cross-sectional study

**DOI:** 10.3389/froh.2022.1041415

**Published:** 2022-12-15

**Authors:** Elizabeth Alpert, Sean G. Boynes, Amber Shaver, Annaliese Cothron

**Affiliations:** ^1^ Department of Restorative Dentistry and Biomaterials Sciences, Harvard School of Dental Medicine, Boston, MA, United States; ^2^ Dental Medicine Consulting, Oakdale, PA, United States; ^3^ American Institute of Dental Public Health, San Antonio, TX, United States; ^4^ Department of Psychology, California State University, Fresno, CA, United States

**Keywords:** COVID-19, oral health, dental care, healthcare finance, underserved populations, Small Business Administration, Paycheck Protection Program, CARES act

## Abstract

**Background:**

The Covid-19 pandemic exacerbated dental staffing shortages, which impact care delivery and ultimately oral health equity. Federal funding efforts like the Paycheck Protection Program (PPP) sought to aid traditionally underserved businesses including those owned by veterans, minority racial and ethnic groups, and women.

**Objectives:**

(1) To examine differences in PPP funding between veteran- and nonveteran-owned dental care delivery businesses and organizations and (2) to analyze other relevant factors associated with variation in PPP funding levels for dental businesses.

**Methods:**

Using publicly available PPP data, we ran unadjusted bivariable and adjusted multivariable linear regression models to estimate associations between loan approval amount and forgiveness amount, veteran status, and relevant covariates.

**Results:**

Minority racial and ethnic groups and women received less PPP funding and less loan forgiveness, on average, compared with non-minority groups. In the adjusted model with no missing self-reported demographic observations at *p* < 0.10, veterans received more PPP funding and loan forgiveness, on average, compared to non-veterans.

**Conclusion:**

To our knowledge, this is the first comprehensive analysis of all dental recipients of PPP funding throughout the United States. Despite PPP program intentions and strategies, traditionally underserved dental businesses did not receive increased funding to support employment.

## Introduction

### Background/rationale

The Covid-19 pandemic impacted the dental care delivery system in multiple domains including education, financing, regulation, and care delivery (1). By April 2020, employment in dental offices plummeted to 44% of pre-pandemic levels. Despite subsequent recovery, significant staffing shortages persist (2). The dental workforce is essential to facilitating oral health access, quality, and cost (3) and therefore to promoting oral health equity.

Congressional action provided multiple mechanisms of addressing dental workforce challenges through federal Covid relief funding: the Economic Injury Disaster Loan (EIDL) small business loan program, American Rescue Plan Act (ARPA) funding, and the Paycheck Protection Program (PPP) (3).

Approximately 9 out of 10 dental private practices utilized the PPP (2), which was established by the Coronavirus Aid, Relief, and Economic Security (CARES) Act and implemented by the U.S. Small Business Administration (SBA). The PPP (4) provided small businesses with funds to pay up to 8 weeks of payroll costs including benefits, as well as interest on mortgages, rent, and utilities. It included provisions for full loan forgiveness (3). After initial program implementation, Congress and the SBA initiated changes to help traditionally underserved businesses receive PPP loans, including those owned by veterans, minority racial and ethnic groups, and women (5).

While there is increased awareness of oral health disparities among veterans (6), current literature does not provide information regarding veteran-owned dental businesses. To promote oral health equity, it is critical to understand dental workforce challenges and impact of federal Covid relief funding among traditionally underserved dental care delivery businesses and organizations.

### Objectives

The first objective of this study was to examine differences in PPP funding between veteran- and nonveteran-owned dental care delivery businesses and organizations. The second objective was to analyze other relevant factors associated with variation in PPP funding for dental businesses besides veteran status. We hypothesized that dental businesses owned by veterans, minorities, and women would receive higher levels of funding and subsequent loan forgiveness, since the SBA implemented program measures to facilitate PPP loans for these groups (5).

## Methods

### Study design and setting

We designed and implemented a cross-sectional study using publicly available PPP loan-level data (7) released by the U.S. Small Business Administration. The data contained all loan approvals over the program duration, from April 3, 2020 (8) to program end on May 31, 2021 (9) and included all U.S. states and territories. The STROBE (Strengthening the Reporting of Observational Studies in Epidemiology) guidelines for reporting observational studies were followed (10).

### Participants and study size

We used a two-step process to select our study sample. First, we identified all possible dental care delivery businesses and organizations in the PPP data through Google search engine optimization keywords for dental businesses (3). We then implemented a filtering approach to eliminate non-dental businesses from the study sample by only keeping observations with North American Industry Classification System (NAICS) codes relevant to dental businesses: (621210 Offices of Dentists; 621111 Offices of Physicians; 621399 Offices of All Other Miscellaneous Health Practitioners; 423450 Medical, Dental, and Hospital Equipment and Supplies Merchant Wholesalers; 339116 Dental Laboratories; and 339114 Dental Equipment and Supplies Manufacturing).

There were no exclusion criteria. After applying the above inclusion criteria, the initial study size was 132,207.

### Variables

#### Primary outcomes

• Loan approval amount in dollars (current)
• Forgiveness amount in dollars

#### Primary exposure

• Veteran status: Veteran, Non-Veteran, Unanswered

#### Covariates

• Race: White, Asian, Black or African American, American Indian or Alaska Native, Other (Eskimo & Aleut, Native Hawaiian or Other Pacific Islander, Puerto Rican, Multi Group), Unanswered
• Ethnicity: Not Hispanic or Latino, Hispanic or Latino, Unanswered
• Gender: Male, Female, Unanswered
• Rurality: Urban, Rural
• Low- and moderate-income indicator: No, Yes
• Number of employees
• Business type: Corporation, Partnership, Sole Proprietorship, Subchapter S Corporation, Limited Liability Partnership, Limited Liability Company, Professional Association, Other
• Loan delivery method: first draw, second draw
• Lender type: Top 5 Bank, Top 5 Alternative Lender, Other

### Data sources/measurement

All data for analysis was obtained from the publicly available PPP loan data and was self-certified by applicants for accuracy (11). Regarding lender type, we used categories previously established in the literature. Top 5 Banks referred to Bank of America, JP Morgan Chase, Wells Fargo, U.S. Bank, or Citibank. Top 5 Alternative Lenders—either fintech banks or lenders supporting major fintech lenders and not traditional depository institutions—included Cross River Bank, Kabbage, Celtic Bank, WebBank, or Customer's Bank (12).

### Statistical methods

Descriptive statistics were used to examine a breakdown of all PPP approved lending for all dental businesses and self-identified veteran-owned dental businesses, along with covariates of interest for each group.

We first looked at the outcome of loan approval amount. Unadjusted bivariable and adjusted multivariable linear regression models were used to estimate the association between loan approval amount and veteran status. Models were adjusted for all covariates. Model 1.A (unadjusted) and Model 2.A (adjusted) analyzed the complete study sample of all dental businesses meeting the specified inclusion criteria. Model 3.A (unadjusted) and Model 4.A (adjusted) contains only dental businesses meeting the specified inclusion criteria and with no missing observations.

We then applied the same approach in evaluating the outcome of forgiveness amount. Model 1.B (unadjusted) and Model 2.B (adjusted) analyzed the complete study sample of all dental businesses meeting the specified inclusion criteria. Model 3.B (unadjusted) and Model 4.B (adjusted) contain only dental businesses meeting the specified inclusion criteria and with no missing observations.

The statistical significance level was set at 10%, and R software version 1.3.1073 was used for analysis.

### Bias

There were similar trends observed in loan size distribution, borrower demographics, business characteristics, and loan characteristics between the entire study sample (*N* = 132,207, all dental businesses meeting the specified inclusion criteria) and the study sample limited to self-identified veteran-owned dental businesses (*N* = 3,495; Tables 1, 2).

**Table 1:** Overview of Payroll Protection Program (PPP) approved lending for dental businesses and loan size distribution, based on veteran status.

	All Dental Businesses		Self-Identified Veteran-Owned Dental Businesses	
	*N* = 132,207		*N* = 3,495	
Loan count	132,207		3,495	
	Net dollars	$11,472,407,083		$312,356,497	
Average loan size	$86,776.09		$89,372.39	
	All Dental Businesses		Self-Identified Veteran-Owned Dental Businesses	
	Net Dollars	Loan Count	Net Dollars	Loan Count
$50K and under	$1,427,125,912	51,145 (38.7%)	$35,503,719	1,191 (34.0%)
>$50K–$100K	$3,382,675,018	46,671 (35.3%)	$97,522,561	1,347 (38.5%)
>$100K–$150K	$2,316,323,611	19,052 (14.4%)	$63,786,695	520 (14.9%)
>$150K–$350K	$2,701,130,620	12,938 (9.8%)	$76,340,030	371 (10.6%)
>$350K–$1M	$1,078,493,970	2,106 (1.6%)	$31,325,576	60 (1.7%)
>$1M–$2M	$316,532,695	224 (0.2%)	$7,877,914	6 (0.2%)
>$2M–$5M	$175,981,398	60 (0.05%)	$0	0
>$5M	$74,143,860	11 (0.008%)	$0	0

Due to rounding, all percentages may not add up to 100.

**Table 2:** Characteristics of study sample and PPP loans, based on veteran status.

	All Dental Businesses				Self-Identified Veteran-Owned Dental Businesses			
	*N* = 132,207				*N* = 3,495			
BORROWER DEMOGRAPHICS								
	Loan count	Net dollars	Average loan size	Average loan size per # of employees	Loan count	Net dollars	Average loan size	Average loan size per # of employees
**Race**								
White	20,772	$1,841,034,166	$88,630.57	$9,484.49	1,604	$141,122,194.40	$87,981.42	$9,832.93
Asian	7,549	$520,122,204	$68,899.48	$8,363.84	332	$22,873,507.60	$68,896.11	$8,088.23
Black or African American	1,291	$87,972,791	$68,143.14	$8,768.34	124	$7,983,254.10	$64,381.08	$8,333.25
American Indian or Alaskan Native	914	$77,356,577	$84,635.20	$9,456.79	64	$6,914,603.40	$108,040.68	$11,680.07
Other*	187	$12,035,065	$64,358.64	$7,438.24	9	$658,487.50	$73,165.28	$10,620.77
Unanswered	101,494	$8,933,886,279	$88,023.79	$9,676.75	1,362	$132,804,449.50	$97,506.94	$9,646.58
**Ethnicity**								
Not Hispanic or Latino	36,949	$3,292,145,925	$89,099.73	$9,395.93	2,385	$214,489,628	$89,932.76	$9,716.40
Hispanic or Latino	3,216	$217,537,421	$67,642.23	$8,183.94	171	$12,139,651	$70,992.11	$9,127.56
Unanswered	92,042	$7,962,723,736	$86,511.85	$9,682.31	939	$85,727,218	$91,296.29	$9,365.00
**Gender**								
Male	41,309	$3,870,433,085	$93,694.67	$9,468.07	2,874	$266,946,465	$92,883.25	$9,629.76
Female	14,765	$1,087,398,752	$73,647.05	$9,269.92	543	$39,390,397	$72,542.17	$9,356.39
Unanswered	76,133	$6,514,575,246	$85,568.35	$9,676.05	78	$6,019,634	$77,174.80	$9,585.41

BUSINESS CHARACTERISTICS								
	Loan count	Net dollars	Average loan size	Average loan size per # of employees	Loan count	Net dollars	Average loan size	Average loan size per # of employees
**Rurality**								
Urban	113,408	$9,823,783,143	$86,623.37	$9,562.54	2,874	$255,587,437	$88,930.91	$9,526.54
Rural	18,799	$1,648,623,940	$87,697.43	$9,582.79	621	$56,769,060	$91,415.56	$9,907.34
**Low- and moderate- income (LMI)**								
No	104,343	$9,095,314,907	$87,167.47	$9,642.56	2,774	$242,738,653	$87,504.92	$9,641.29
Yes	27,863	$2,377,067,210	$85,312.68	$9,281.43	721	$69,617,844	$96,557.34	$9,430.76
**Number of employees**								
0 to 4	36,686	$1,016,189,055	$27,699.64	$10,791.24	841	$24,960,856	$29,679.97	$10,735.85
5 to 9	56,431	$3,745,369,267	$66,370.78	$9,782.56	1,559	$104,649,255	$67,125.88	$9,778.48
10 to 19	30,781	$3,831,451,904	$124,474.58	$9,747.11	863	$108,229,098	$125,410.31	$9,910.18
20 to 49	6,997	$1,880,870,952	$268,811.06	$9,740.25	198	$53,489,903	$270,151.03	$10,176.92
50 to 99	892	$475,144,908	$532,673.66	$7,993.29	29	$18,348,863	$632,719.43	$8,834.31
100 to 499	405	$481,519,342	$1,188,936.64	$6,958.57	1	$68,602	$68,602.00	$137.20
500+	15	$41,861,657	$2,790,777.13	$5,581.55	4	$2,609,919	$652,479.75	$3,354.65
**Business type**								
Corporation	54,799	$4,856,578,694	$88,625.32	$9,590.08	1,485	$136,987,019	$92,247.15	$9,317.58
Partnership	2,309	$321,153,500	$139,087.70	$9,590.08	52	$7,241,897	$139,267.24	$10,044.24
Sole Proprietorship	11,020	$552,818,607	$50,165.03	$9,357.59	322	$18,583,073	$57,711.41	$9,932.16
Subchapter S Corporation	28,338	$2,547,570,096	$89,899.43	$9,831.70	696	$63,070,280	$90,618.22	$10,000.04
Limited Liability Partnership (LLP)	1,091	$137,442,989	$125,978.91	$8,753.77	31	$5,795,220	$186,942.58	$10,517.64
Limited Liability Company (LLC)	30,920	$2,721,594,638	$88,020.53	$9,222.68	803	$69,296,265	$86,296.72	$9,541.00
Professional Association	2,403	$242,013,547	$100,713.09	$10,409.19	86	$8,666,152	$100,769.21	$10,018.67
Other	1,327	$93,235,012	$70,259.99	$10,452.36	20	$2,716,591	$135,829.54	$9,736.88

LOAN CHARACTERISTICS								
	Loan count	Net dollars	Average loan size	Average loan size per number of employees	Loan count	Net dollars	Average loan size	Average loan size per number of employees
**Loan delivery method**								
First draw	82,512	$7,009,869,400	$84,955.76	$9,626.92	1,998	$169,907,915	$85,039.00	$9,537.89
Second draw	49,695	$4,462,537,683	$89,798.52	$9,470.44	1,497	$142,448,582	$95,156.03	$9,660.81
**Lender type**								
Top 5 Bank	29,092	$1,945,978,909	$66,890.52	$8,164.65	602	$45,083,945	$74,890.27	$7,927.54
Top 5 Alternative Lender	7,745	$425,881,998	$54,987.99	$9,042.47	134	$8,507,660	$63,490.00	$9,108.84
Other	95,370	$9,100,546,175	$95,423.57	$9,957.71	2,759	$258,764,892	$93,789.38	$9,976.29

*“Other” includes the following: Eskimo & Aleut, Native Hawaiian or Other Pacific Islander, Puerto Rican, Multi Group.

There were many unanswered responses to the borrower demographic questions (Table 2). Among all dental businesses (*N* = 132,207), 64.4% did not identify their veteran status (*N* = 85,245). 76.8% did not answer race (*N* = 101,494), 69.6% did not identify their ethnicity (*N* = 92,042), and 57.6% did not specify their gender (*N* = 76,133). To address this, we ran unadjusted bivariable and adjusted multivariable linear regression models to estimate the associations between veteran status and loan approval amount and veteran status and forgiveness amount with both the entire study sample (*N* = 132,207) and with the study sample restricted to dental businesses with no missing demographic observations (*N* = 23,355; Tables 3, 4). Generally, findings were consistent between the models with and without the missing data.

**Table 3:** Associations between payroll protection program (PPP) loan approval amount and veteran status and other covariates.

	Model 1.A *N* = 132,207	Model 2.A *N* = 132,207	Model 3.A *N* = 23,355	Model 4.A *N* = 23,355
	Unadjusted (95% CI)	Adjusted (95% CI)	Unadjusted (95% CI)	Adjusted (95% CI)
**Veteran status**				
Non-Veteran	REF	REF	REF	REF
Veteran	1,907 (−2,665, 6,479)	1,280 (−4,173, 6,733)	3,416 (−1,246, 8,078)	5,032. (−405, 10,469)
Unanswered	−1,147 (−2,680, 385)	1,223 (−2,863, 5,309)		
**Race**				
White		REF		REF
Asian		−9,370 *** (−12,516, −6,225)		−11,527 *** (−14,387, −8,668)
Black or African American		−6,712 * (−13,117, −307)		−8,176 ** (−13,927, −2,424)
American Indian or Alaskan Native		−7,738 (−24,104, 8,627)		−7,611 (−24,651, 9,428)
Other		−15,299. (−30,705, 107)		−12,348. (−26,388, 1,692)
Unanswered		6 (−3,148, 3,161)		
**Ethnicity**				
Not Hispanic or Latino		REF		REF
Hispanic or Latino		−12,293 *** (−16,698, −7,889)		−13,506 *** (−18,035, −8,976)
Unanswered		1,240 (−2,022, 4,502)		
**Gender**				
Male		REF		REF
Female		−6,836 *** (−9,500, −4,172)		−7,454 *** (−10,179, −4,729)
Unanswered		−10,139 *** (−14,334, −5,943)		
**Rurality**				
Urban		REF		REF
Rural		14,109 *** (9,587, 18,630)		17,316 *** (9,166, 25,465)
**Low- and moderate- income (LMI)**				
No		REF		REF
Yes		−1,571. (−3,357, 215)		944 (−2,108, 3,996)
**Number of employees**		3,050 *** (2,999, 3,101)		1,967 *** (1,872, 2,062)
**Business type**				
Limited Liability Corporation (LLC)		REF		REF
Corporation		1,702 (−326, 3,730)		3,054. (−404, 6,513)
Limited Liability Partnership (LLP)		33,631 *** (23,599, 43,663)		14,249 (−5,959, 34,457)
Partnership		17,950 *** (10,846, 25,054)		44,110 *** (29,651, 58,578)
Professional association		838 (−4,561, 6,237)		2,332 (−5,449, 10,113)
Sole proprietorship		−16,407 *** (−19,694, −13,120)		−14,974 *** (−21,287, −8,660)
S corp		2,750 * (277, 5,224)		−86 (−4,619, 4,448)
**Loan delivery method**				
Payroll Protection Program (PPP) for first draw		REF		REF
Positive Payment System (PPS) for second draw		2,892 *** (1,302, 4,482)		−711 (−3,329, 1,906)
**Lender type**				
Top 5 bank		REF		REF
Top 5 alternative lender		−4,626 *** (−6,528, −2,725)		−2,907 (−6,735, 921)

Signif. codes: 0 “***” 0.001 “**” 0.01 “*” 0.05 “.” 0.1 “ ” 1.

Linear regression:

Model 1.A contains all dental businesses, unadjusted.

Model 2.A contains all dental businesses, adjusted for all significant covariates from bivariate analysis plus veteran status and rurality.

Model 3.A contains only dental businesses with no missing demographic observations, unadjusted.

Model 4.A contains only dental businesses with no missing demographic observations, adjusted for all significant covariates from bivariate analysis plus veteran status and rurality.

**Table 4:** Associations between paycheck protection program (PPP) loan forgiveness amount and veteran status and other covariates.

	Model 1.B *N* = 132,207	Model 2.B *N* = 132,207	Model 3.B *N* = 23,355	Model 4.B *N* = 23,355
	Unadjusted (95% CI)	Adjusted (95% CI)	Unadjusted (95% CI)	Adjusted (95% CI)
**Veteran status**				
Non-Veteran	REF	REF	REF	REF
Veteran	1,300 (−3,192, 5,793)	2,246 (−3,121, 7,614)	3,925 (−780, 8,630)	5,251. (−160, 10,663)
Unanswered	−1,068 (−2,577, 442)	1,240 (−2,829, 5,309)		
**Race**				
White		REF		REF
Asian		−9,139 *** (−12,232, −6,046)		−11,380 *** (−14,217, −8,543)
Black or African American		−6,473 * (−12,923, −24)		−8,114 ** (−13,932, −2,295)
American Indian or Alaskan Native		−6,932 (−23,363, 9,498)		−7,656 (−24,905, 9,592)
Other		−13,176. (−28,852, 2,500)		−10,582 (−24,919, 3,755)
Unanswered		38 (−3,069, 3,146)		
**Ethnicity**				
Not Hispanic or Latino		REF		REF
Hispanic or Latino		−13,357 *** (−17,702, −9,011)		−14,655 *** (−19,170, −10,139)
Unanswered		406 (−2,818, 3,631)		
**Gender**				
Male		REF		REF
Female		−7,597 *** (−10,218, −4,976)		−7,964 *** (−10,666, −5,262)
Unanswered		−9,917 *** (−14,096, −5,737)		
**Rurality**				
Urban		REF		REF
Rural		13,922 *** (9,429, 18,414)		16,739 *** (8,620, 24,857)
**Low- and moderate- income (LMI)**				
No		REF		REF
Yes		−1,733. (−3,498, 31)		−240 (−3,279, 2,800)
**Number of employees**		2,936 *** (2,887, 2,986)		1,991 *** (1,897, 2,086)
**Business type**				
Limited Liability Corporation (LLC)		REF		REF
Corporation		1,747. (−251, 3,745)		3,072. (−352, 6,497)
Limited Liability Partnership (LLP)		36,049 *** (26,225, 45,874)		16,318 (−3,544, 36,179)
Partnership		19,768 *** (12,806, 26,730)		46,949 *** (32,520, 61,379)
Professional association		989 (−4,292, 6,269)		3,130 (−4,502, 10,762)
Sole proprietorship		−16,129 *** (−19,365, −12,893)		−14,509 *** (−20,767, −8,262)
S corporation		2,756 * (316, 5,196)		255 (−4,259, 4,768)
**Loan delivery method**				
First draw		REF		REF
Second draw		3,235 *** (1,651, 4,820)		507 (−2,103, 3,118)
**Lender type**				
Top 5 bank		REF		REF
Top 5 alternative lender		−3,190 ** (−5,096, −1,284)		−746 (−4,645, 3,153)

Signif. codes: 0 “***” 0.001 “**” 0.01 “*” 0.05 “.” 0.1 “ ” 1.

Linear regression:

Model 1.B contains all dental businesses, unadjusted.

Model 2.B contains all dental businesses, adjusted for all significant covariates from bivariate analysis plus veteran status and rurality.

Model 3.B contains only dental businesses with no missing demographic observations, unadjusted.

Model 4.B contains only dental businesses with no missing demographic observations, adjusted for all significant covariates from bivariate analysis plus veteran status and rurality.

Additionally, the publicly released PPP data was limited to loan approvals and did not include rejected or canceled loans. These applicants' demographic, business, and loan characteristics may exhibit differences compared with those of approved applicants.

## Results

### Participants

The complete study sample consisted of 132,207 loans distributed to dental businesses. 3,495 of those had owners who self-identified as veterans. There were 23,355 businesses that provided complete demographic information (veteran status, race, ethnicity, and gender).

### Outcome data

Average loan size was $86,776 among all dental businesses and $89,372 among self-identified veteran-owned dental businesses. Nearly three-quarters of loans were $100,000 and under (74% among all dental businesses and 72.5% among veteran-owned dental businesses). Almost 90% of loans were $150,000 and under (88.4% for all businesses and 87.4% among veteran-owned). For loans above $1,000,000, there were 295 loans (0.2%) among all businesses and 6 loans (0.2%) among veteran-owned (Table 1).

Looking at demographic characteristics among veteran-owned businesses, average loan size per number of employers was $9,833 among white borrowers and $8,333 among Black borrowers. Loan size was $9,716 among non-Hispanic or Latino borrowers, compared with $9,128 for Hispanic or Latino borrowers. It was higher for males ($9,630) than females ($9,356) (Table 2).

### Main results

Among the unadjusted models, neither Model 1.A (with all observations) nor Model 3.A (with no missing demographic observations) demonstrated significant associations between veteran status and loan amount. Adjusted Model 2.A (with all observations) showed significant associations between loan amount and race, ethnicity, gender, rurality, low- and moderate-income-indicator, number of employees, business type, loan delivery method, and lender type. Adjusted Model 4.A (with all observations) showed significant associations between loan amount and race, ethnicity, gender, rurality, number of employees, and business type (Table 3).

There were similar findings with the outcome of forgiveness amount. Neither of the unadjusted models – Model 1.B (with all observations) and Model 3.B (with no missing observations) – showed a significant relationship between veteran status and loan forgiveness amount. However, adjusted Model 2.B (with all observations) demonstrated significant associations between forgiveness amount and race, ethnicity, gender, rurality, low- and moderate-income indicator, number of employees, loan delivery method, business type, and lender type. Adjusted Model 4.B (with no missing demographic observations) had significant relationships between forgiveness amount and veteran status, race, ethnicity, gender, rurality, number of employees, and business type (Table 4).

## Discussion

### Key results and interpretation

Our initial hypothesis that dental businesses owned by veterans, minorities, and women would receive the most PPP funding was weakly supported. Among the study sample without missing demographic information (*N* = 23,355), veteran-owned dental businesses received $5,032 (*p* = 0.07) more, on average, and had an additional $5,251 forgiven (*p* = 0.06) compared with non-veteran-owned businesses. While this was not significant at the *p* < 0.05 level, it does provide some evidence for the PPP intention to assist veteran-owned businesses. However, opposite conclusions were made regarding minority- and women-owned businesses.

After adjusting for relevant covariates in both the entire study sample (*N* = 132,207) and study sample with complete demographic information (*N* = 23,355), there were significant associations between loan amount and race, ethnicity, gender, rurality, number of employees, and business type.

Compared with white-owned businesses, dental businesses owned by Asian, Black or African American, and Other (Eskimo & Aleut, Native Hawaiian or Other Pacific Islander, Puerto Rican, Multi Group) racial groups both received less PPP funding and had smaller amounts forgiven. This echoes other literature that has already demonstrated evidence of disparate lending (12). Hispanic and Latino-owned businesses were also given smaller amounts and had smaller amounts of loans forgiven, as were female-owned businesses. These findings question the effectiveness of SBA-implemented strategies to fund traditionally underserved businesses, which included more diversity in lender type and efforts to target funding to these groups (5). Among the entire sample, those with a top 5 alternative lender had $4,626 less in PPP loan amount and had $3,190 less forgiven compared to those from a top 5 bank. This queries the role of nontraditional lender types, who may be more accessible to underserved groups.

The discrepancy in granted loan amounts and loan forgiveness among racial and ethnic minorities and women may have detrimental implications on business viability. While some studies indicated limited impact on long term outcomes, more evidence reinforces negative impacts within the pattern of lending discrepancy (13–15). Even when minority and women business owners receive loans, they often come with more restrictive terms and higher rates compared to their white male counterparts (14, 16–18). Small business who are refused loans, are only allowed limited funding, or have smaller amounts of loan forgiveness may experience financial uncertainty as a result. Findings of this study indicating smaller amounts of loan forgiveness convey alarming implications as minority and women business owners will experience higher burdens of loan debt, reducing profit margins and jeopardizing sustainability. Evidence suggests that minority small business owners, especially Black business owners, already distrust lending systems and may not seek loans due to perceived discrimination (16). The results of this study may validate that attitude increasing the negative perception of banking institutions. Ultimately, these data cannot derive causation into the disparate lending and forgiveness amounts which requires additional exploration.

Rural businesses secured more funding and increased forgiven amounts, on average, compared with urban businesses. These implications are important for the dental workforce as rural areas were most impacted by Covid-related staffing and revenue reductions (1, 3). While the PPP appeared to sustain many dental rural practices during the early period of the pandemic, rural areas and Medicaid provider networks experienced the most significant attrition of workforce and active employment since 2020 (3, 19). Previous research determined that the PPP had a limited effect on employment (20). Rural communities have an older dentist population and an increased number of dentists retiring. Rural dental healthcare workers also report lower levels of satisfaction with their job or career choice which may also contribute to staffing shortages and limiting the effect of the PPP for rural dental practices (3, 21, 22). Further research is warranted to better understand the continued attrition of dental care teams in rural communities.

### Limitations

As discussed, there were many missing self-reported demographic characteristics, although information was provided regarding business and loan characteristics. Within the initial loan application form, the SBA did not ask for any demographic data from PPP applicants (23). This is not traditional SBA practice, and it leaves the collection of this vital data up to the individual lender, which resulted in this information not being collected routinely (24). Additionally, the racial categories do not allow for the disaggregation into specific and critical sub-groups (23, 24). Selection bias may have impacted reports of veteran status, race, ethnicity, and gender. Individuals with all reported demographics may not be reflective of the entire study population. However, similar trends in loan approval amounts and loan forgiveness amounts were observed between the entire group and those without any missing observations (Tables 3, 4).

Additionally, PPP data did not include canceled or rejected loans, so it was not possible to evaluate the role of veteran status, race, ethnicity, gender, business characteristics, or loan characteristics, in that context.

For a more comprehensive assessment of dental recipients of pandemic relief funding, future analyses should examine additional federal funding mechanisms, including the Economic Injury Disaster Loan (EIDL) small business loan program, and explore outcomes on the region, state, and county levels. Combining PPP data with other data sources would allow comparisons of PPP funding outcomes like oral health utilization, emergency department use for non-traumatic dental problems, and other measures of oral health equity. Future research should also explore the long-term financial sustainability among dental care delivery sites that received PPP funds, particularly with respect to veteran, minority, women, and rural business owners.

### Generalizability

To our knowledge, this is the first comprehensive analysis of all dental recipients of PPP funding throughout the United States. These findings contribute to the limited body of literature on veteran-owned dental businesses and adds evidence on disparate lending in the PPP.

## Data Availability

Publicly available datasets were analyzed in this study. This data can be found here: https://data.sba.gov/dataset/ppp-foia.
